# Assessment of Racial and Ethnic Disparities in the Use of Medication to Treat Opioid Use Disorder Among Pregnant Women in Massachusetts

**DOI:** 10.1001/jamanetworkopen.2020.5734

**Published:** 2020-05-26

**Authors:** Davida M. Schiff, Timothy Nielsen, Bettina B. Hoeppner, Mishka Terplan, Helena Hansen, Dana Bernson, Hafsatou Diop, Monica Bharel, Elizabeth E. Krans, Sabrina Selk, John F. Kelly, Timothy E. Wilens, Elsie M. Taveras

**Affiliations:** 1 Division of General Academic Pediatrics, MassGeneral Hospital for Children, Boston, Massachusetts; 2 Child Population and Translational Health Research, University of Sydney, Randwick, New South Wales, Australia; 3 Department of Psychiatry, Massachusetts General Hospital, Boston; 4 Friends Research Institute, Baltimore, Maryland; 5 Department of Psychiatry, New York University, New York, New York; 6 Massachusetts Department of Public Health, Boston; 7 Department of Obstetrics, Gynecology, and Reproductive Sciences, Magee Women’s Hospital, University of Pittsburgh, Pittsburgh, Pennsylvania; 8 Division of Child and Adolescent Psychiatry, Massachusetts General Hospital, Boston

## Abstract

**Importance:**

Racial and ethnic disparities persist across key health and substance use treatment outcomes for mothers and infants. The use of medications, such as methadone or buprenorphine, for the treatment of opioid use disorder (OUD) has been associated with improvements in the outcomes of mothers and infants; however, only half of all pregnant women with OUD receive these medications. The extent to which maternal race or ethnicity is associated with the use of medication to treat OUD, the duration of the use of medication to treat OUD, and the type of medication used to treat OUD during pregnancy are unknown.

**Objective:**

To examine the extent to which maternal race and ethnicity is associated with the use of medications for the treatment of OUD in the year before delivery among pregnant women with OUD.

**Design, Setting, and Participants:**

This retrospective cohort study used a linked population-level statewide data set of pregnant women with OUD who delivered a live infant in Massachusetts between October 1, 2011, and December 31, 2015. Of 274 234 total deliveries identified, 5247 deliveries among women with indicators of having OUD were included in the analysis. Maternal race and ethnicity were defined as white non-Hispanic, black non-Hispanic, or Hispanic based on self-reported data on birth certificates.

**Main Outcomes and Measures:**

Main outcomes were the receipt of any medication for OUD, the consistency of the use of medication (at least 6 continuous months of use before delivery, inconsistent use, or no use) for the treatment of OUD, and the type of medication (methadone or buprenorphine) used to treat OUD. Multivariable models were adjusted for maternal sociodemographic characteristics, comorbidities, and any significant interactions between the covariates and race and ethnicity.

**Results:**

The sample included 5247 pregnant women with OUD who delivered a live infant in Massachusetts during the study period. The mean (SD) maternal age at delivery was 28.7 (5.0) years; 4551 women (86.7%) were white non-Hispanic, 462 women (8.8%) were Hispanic, and 234 women (4.5%) were black non-Hispanic. A total of 3181 white non-Hispanic women (69.9%) received any type of medication for the treatment of OUD in the year before delivery compared with 228 Hispanic women (49.4%) and 108 black non-Hispanic women (46.2%). Compared with white non-Hispanic women, black non-Hispanic and Hispanic women had a substantially lower likelihood (adjusted odds ratio [aOR], 0.37; 95% CI, 0.28-0.49 and aOR, 0.42; 95% CI, 0.35-0.52, respectively) of receiving any medication for the treatment of OUD. Stratification by maternal age identified greater disparities among younger women. Black non-Hispanic and Hispanic women also had a lower likelihood (aOR, 0.24; 95% CI, 0.17-0.35 and aOR, 0.34; 95% CI, 0.27-0.44, respectively) of consistent use of medication for the treatment of OUD compared with white non-Hispanic women. With respect to the type of medication used to treat OUD, black non-Hispanic and Hispanic women had a lower likelihood (aOR, 0.60; 95% CI, 0.40-0.90 and aOR, 0.77; 95% CI, 0.58-1.01, respectively) than white non-Hispanic women of receiving buprenorphine treatment compared with methadone treatment.

**Conclusions and Relevance:**

This study found racial and ethnic disparities in the use of medications to treat OUD during pregnancy, with black non-Hispanic and Hispanic women significantly less likely to use medications consistently or at all compared with white non-Hispanic women. Further investigation of patient, clinician, treatment program, and system-level factors associated with these findings is warranted.

## Key Points

**Question:**

Do differences by maternal race and ethnicity exist in the use of methadone or buprenorphine medications for the treatment of opioid use disorder during pregnancy?

**Findings:**

In this cohort study of 5247 women with opioid use disorder who delivered a live infant, black non-Hispanic and Hispanic women with opioid use disorder were significantly less likely to use any medication for treatment and were less likely to consistently use medication for treatment during pregnancy compared with white non-Hispanic women with opioid use disorder.

**Meaning:**

This study found racial and ethnic disparities in the use of medications for the treatment of opioid use disorder during pregnancy among a large population-level sample of women with opioid use disorder in Massachusetts; further investigation is warranted to explore the factors associated with inequitable access to and receipt of medication.

## Teaser

This cohort study assesses the extent to which maternal race and ethnicity is associated with the use of medications for the treatment of opioid use disorder in the year before delivery among pregnant women with opioid use disorder in Massachusetts.

## Introduction

The number of pregnant women with opioid use disorder (OUD) has increased 4-fold during the past decade, paralleling the increase of opioid use in the general population in the United States.^1^ During pregnancy, medication for the treatment of OUD (defined as buprenorphine or methadone) combined with behavioral therapy is the recommended management for women with OUD.^2,3,4^ The use of medications for the treatment of OUD has been associated with improvements in prenatal care adherence and pregnancy outcomes, including lower rates of preterm birth and low birth weight and reductions in maternal relapse and overdose.^2,5,6,7^ Although pregnancy provides a motivational opportunity for women with OUD to initiate treatment with medication to engage in care and increase their engagement with health care services,^8^ only 50% to 60% of pregnant women with OUD use any medication for the treatment of OUD.^9,10,11^

The underuse of medication for the treatment of OUD in the general population has been attributed to a shortage of treatment programs and clinicians offering medications for OUD, insufficient insurance coverage of services, and persistent stigma and misunderstanding about the use of medications for the treatment of OUD.^12,13,14,15,16,17^ In addition, racial and ethnic disparities in the use of medications to treat OUD have been described, including differential access to buprenorphine prescribers by neighborhood,^18^ greater buprenorphine prescription rates for white non-Hispanic individuals compared with black non-Hispanic individuals,^19^ and less timely receipt of medication to treat OUD among black youths compared with white youths.^20^

The prenatal period offers an opportunity to assess disparities in treatment use, as most pregnant individuals are eligible for health insurance, federal regulations emphasize priority access to addiction treatment during pregnancy, and the use of medication for the treatment of OUD (in contrast with medically assisted withdrawal from opioids) is recommended by all professional societies and public health agencies.^2,3^ To our knowledge, 2 studies have examined the use of medications to treat OUD by race among pregnant women with OUD. Among a cohort of Medicaid enrollees in Pennsylvania, pregnant women of color were reported to be less likely to receive any medication to treat OUD, and among women receiving buprenorphine treatment, women of color had higher rates of early discontinuation and decreasing adherence to buprenorphine treatment during pregnancy compared with white non-Hispanic women.^9,21^ However, it remains uncertain if racial and ethnic health disparities persist after adjusting for health status.^22,23^ Furthermore, health inequities, defined as disparities that are both preventable and unjust, have been identified across other key maternal health outcomes, including prenatal care engagement, maternal mortality, and preterm infant birth.^24,25,26^ Understanding at what point along the OUD treatment cascade (which includes OUD diagnosis, engagement in care, treatment use, and adherence in treatment)^27^ racial and ethnic disparities may be present is an important first step to addressing potential inequities in the use of medication for the treatment of OUD.

Therefore, the objective of our study was to explore the extent to which maternal race or ethnicity was associated with (1) any use of medication to treat OUD during pregnancy, (2) the duration of the use of medication to treat OUD during pregnancy, and (3) the type of medication used to treat OUD. Data were obtained from a population-level linked public health data set of women with OUD who delivered a live infant in Massachusetts. We hypothesized that, among women with OUD, white non-Hispanic women would be more likely to receive any medication for the treatment of OUD, more likely to consistently use medication to treat OUD during pregnancy, and more likely to receive buprenorphine treatment compared with their black non-Hispanic and Hispanic counterparts.

## Methods

### Design

We performed a retrospective analysis of a cohort identified through the Public Health Data Warehouse, which is a linked statewide data set. This data set was established as part of a Massachusetts legislative mandate and is overseen by the Massachusetts Department of Public Health.^28,29,30^ Between 2011 and 2015, the Massachusetts Department of Public Health linked a variety of state data sets, including vital records, the all-payer claims database, state-funded addiction treatment data from the Bureau of Substance Addiction Services, acute care hospital records (including inpatient hospitalization, outpatient observation, and emergency department discharge data), and data from the prescription monitoring program. A full description of the data sets linked, the data structure, and the linkage rates across data sets has been previously described.^31^ This study followed the Strengthening the Reporting of Observational Studies in Epidemiology (STROBE) reporting guideline for cohort studies. The institutional review board of Partners HealthCare reviewed this study and deemed it non–human subjects research that was exempt from the need for informed consent.

### Participants

We identified Massachusetts residents who delivered a live infant with a documented gestational age of 20 weeks or more in Massachusetts using birth certificate data. Each participant’s individual pregnancy period was calculated based on gestational age at delivery. We included women who delivered an infant between October 1, 2011, and December 31, 2015, to allow for 9 months of treatment data before delivery. Data on fetal deaths were not available. Singleton and multiple births were included, with multiple births treated as a single delivery episode. When a person had multiple deliveries during the study period, only the first delivery in the period was included. The birth certificate linkage rate with the main data set (ie, the all-payer claims database) for our study period was 91.7%.

Next, we restricted our sample to women with indicators of having OUD and clinical indications for methadone or buprenorphine treatment during pregnancy. An indicator of having OUD during pregnancy was defined as any one of the following: (1) a diagnosis of OUD from hospital discharge or all-payer claims database records (using *International Classification of Diseases, Ninth Revision, Clinical Modification* codes); (2) an opioid overdose event, defined as a claims diagnosis for opioid overdose or an ambulance encounter for opioid overdose; (3) enrollment in a state-funded treatment program for an opioid problem, including acute treatment services, crisis stabilization services, residential programs, and intensive outpatient programs; (4) receipt of methadone or buprenorphine treatment; or (5) an insurance claim for an infant diagnosis of neonatal abstinence syndrome (NAS; eTable 1 in the Supplement). Mothers who were identified solely by an infant diagnosis of NAS were excluded if they had any opioid prescription in the 3 months before delivery or if their child was born at or before 34 weekss gestation to prevent misclassification of women with chronic pain and iatrogenic cases of NAS, respectively. If a person was identified as having OUD from a diagnosis claim alone (without criteria 2-5), she was excluded owing to no identifiable measure of clinical need for medication to treat OUD during pregnancy (eTable 2 and eTable 3 in the Supplement).

### Outcomes

Our main outcomes were the use of any medication for the treatment of OUD, the extent of medication used to treat OUD, and the type of medication used to treat OUD. Because treatment data were reported monthly, any use of medication to treat OUD was defined as the receipt of buprenorphine or methadone treatment, starting with the month of conception and ending with the month of delivery. Data on the use of medication to treat OUD were identified from the following: (1) insurance claims for methadone treatment (Healthcare Common Procedure Coding System code H0020), (2) receipt of methadone treatment from state-funded treatment programs, and (3) filled prescriptions for buprenorphine or buprenorphine/naloxone identified from prescription monitoring program data. Naltrexone was not included in our definition of medication to treat OUD because it is not currently recommended for use during pregnancy.

The extent of the use of medication to treat OUD was defined as consistent (monthly medication to treat OUD that was measured for at least 6 months of treatment before delivery to estimate the number of women receiving treatment throughout their second and third trimesters), inconsistent (any medication to treat OUD in the year before delivery but with gaps in treatment months), and no medication (no indication of the receipt of methadone or buprenorphine treatment). The type of medication used to treat OUD was categorized as buprenorphine, methadone, or neither. Deliveries among women who received both buprenorphine and methadone therapies were classified as methadone, as most individuals transition from buprenorphine to methadone treatment when clinically indicated.

### Exposures

Our primary exposure was maternal race and ethnicity, documented from self-reported birth certificate records. If race and ethnicity data were missing on the birth certificate (1.2% of deliveries) but available across the linked data set, that value was included (accounting for 0.7% of deliveries). Women were categorized as white non-Hispanic, black non-Hispanic, Hispanic, or other race/ethnicity. Multiracial individuals were assigned to the self-reported racial classification with the smallest total representation in the general population. Other races represented 1% of the sample and were excluded owing to small sample sizes. Additional maternal demographic variables included age at delivery, highest educational level, enrollment in Medicaid (MassHealth) during the month of delivery, marital status, and geographic location of residence (categorized as either rural or nonrural based on total population, density, census tract, or hospital licensure).

Psychosocial and health care use characteristics included a maternal diagnosis of anxiety or depression during pregnancy, an opioid prescription (excluding buprenorphine) that was filled in the 3 months before delivery (excluding delivery month), incarceration (release from a Massachusetts prison or jail during the study period), homelessness (during the study period), high use of unscheduled care (≥3 emergency department and/or obstetric triage visits during pregnancy), and adequacy of prenatal care use (using the Kotelchuck Index from the birth certificate).^32^

### Statistical Analysis

We used descriptive statistics to compare the characteristics of our cohort by race/ethnicity and level of treatment engagement. Fisher exact and χ^2^ tests were performed to compare across groups. In our multivariable models, we used statistical model-building criteria (ie, *P* < .05 for both race/ethnicity and any of the 3 medications for OUD outcomes; eTable 4 and eTable 5 in the Supplement) for their inclusion. Based on these criteria, we included age, rural residence, emergency department service use, and opioid prescriptions in the last 3 months of pregnancy. In addition, we included education and health insurance as an adjustment for socioeconomic status and maternal diagnosis of anxiety and/or depression as an adjustment for the association of mental health conditions and treatment receipt.

First, we used logistic regression analysis to estimate the strength of the association between maternal race/ethnicity and any use of medication to treat OUD. Second, we used nominal logistic regression to examine the extent of treatment use and the association with maternal race/ethnicity. We compared consistent and inconsistent use of medication with no use of medication for the treatment of OUD. Next, using nominal logistic regression analysis, we compared buprenorphine treatment with methadone treatment and buprenorphine treatment with no treatment. To assess the association of race/ethnicity with treatment engagement, we removed race/ethnicity and kept other maternal covariates in our model to calculate a pseudo-*R*^2^ value before and after treatment using Nagelkerke mirrors.^33^ We assessed the significance of the interaction of race/ethnicity and all included covariates and retained significant interaction terms in the final model. When a significant interaction was identified, separate effect measures were presented for each level of the relevant covariate.

For the sensitivity analyses, we first performed an analysis excluding black non-Hispanic and Hispanic women to address the potential of a differential diagnosis of NAS by maternal and infant race/ethnicity because these women were more likely to be identified as having OUD by NAS diagnosis alone. Second, we performed an analysis including all individuals who had an OUD diagnosis code but had been excluded (no indication of a clinical need for medication to treat OUD). Third, to determine whether the group of individuals receiving both methadone and buprenorphine therapies differed, we performed an analysis excluding this group.

## Results

### Demographic Characteristics

**Figure 1.**
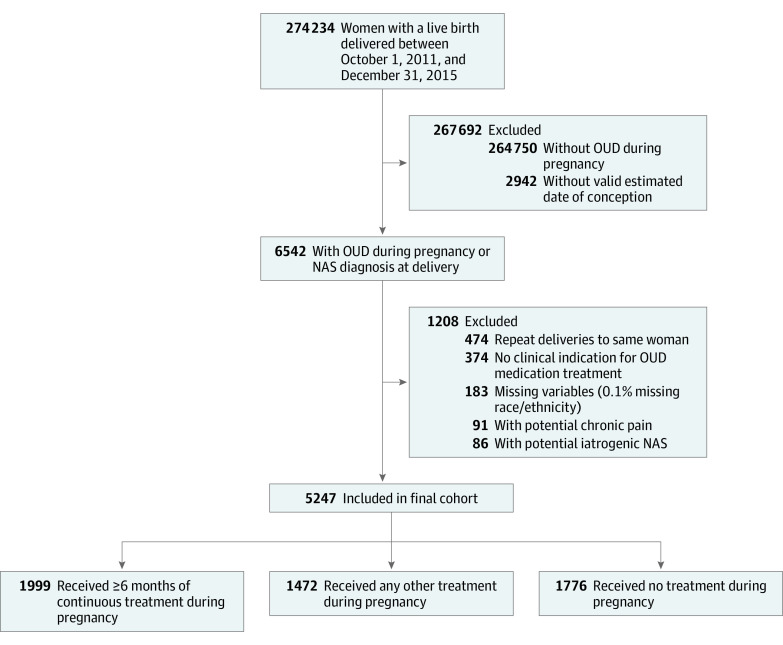
Study Flowchart NAS indicates neonatal abstinence syndrome, and OUD, opioid use disorder.

Of 274 234 deliveries in Massachusetts resulting in a live birth, we identified 5247 deliveries to women with indicators of having OUD, after excluding deliveries owing to possible iatrogenic NAS, other race/ethnicity, no identified clinical need for medication to treat OUD, multiple deliveries in the study period, and missing variables (Figure 1). The mean (SD) age of all participants was 28.7 (5.0) years. Among deliveries to women with OUD, 4551 women (86.7%) were white non-Hispanic, 462 women (8.8%) were Hispanic, and 234 women (4.5%) were black non-Hispanic compared with all deliveries (n = 271 292) with a valid estimated date of conception in Massachusetts between October 2011 and December 2015, of which 170 716 women (62.9%) were white non-Hispanic, 46 901 women (17.3%) were Hispanic, and 24 989 women (9.2%) were black non-Hispanic. Compared with white non-Hispanic women, black non-Hispanic and Hispanic women were older, had lower educational levels, were more likely to live in an urban area, had higher unscheduled emergency department use, were less likely to receive an opioid prescription in the 3 months before delivery, had lower use of publicly funded addiction programs, and were more likely to be identified in the cohort by an infant diagnosis of NAS alone (Table 1). Black non-Hispanic and Hispanic women also had lower rates of exclusive use of buprenorphine treatment.

**Table 1.** Characteristics of Pregnant Women With Opioid Use Disorder by Race/Ethnicity^a^

Characteristic	No. (%) (N = 5247)			*P* value
	White non-Hispanic (n = 4551)	Black non-Hispanic (n = 234)	Hispanic (n = 462)	
Demographic characteristics				
Age, y				
≤25	1283 (28.2)	71 (3.3)	136 (29.4)	.02
26-34	2682 (58.9)	123 (52.6)	247 (52.6)	
≥35	586 (12.9)	40 (17.1)	79 (17.1)	
Educational level				
High school or less	2385 (52.4)	127 (54.3)	300 (64.9)	<.001
Some college or more	2166 (47.6)	107 (45.7)	162 (35.1)	
Enrollment in Medicaid (MassHealth) during the month of delivery	4065 (89.3)	215 (91.9)	426 (92.2)	.08
Married	800 (17.6)	36 (15.4)	78 (16.9)	.66
Rural vs urban residence at time of delivery	512 (11.3)	NA^b^	19 (4.1)	<.001
Psychosocial characteristics and health care use during pregnancy				
Anxiety diagnosis	1135 (24.9)	47 (2.1)	105 (22.7)	.16
Depression diagnosis	1270 (27.9)	63 (26.9)	149 (32.3)	.13
Any opioid prescription in last 3MD (excluding buprenorphine)	172 (3.8)	NA^b^	NA^b^	.01
Incarcerated in prison or jail^c^	773 (17.0)	41 (17.5)	62 (13.4)	.14
Homeless^c^	1067 (23.5)	70 (29.9)	118 (25.5)	.05
≥3 ED visits	798 (17.5)	58 (24.8)	86 (18.6)	.02
Adequacy of prenatal care				
Less than adequate	1884 (41.4)	103 (44.0)	215 (46.5)	.16
Adequate	1257 (27.6)	65 (27.8)	107 (23.2)	
Intensive	1410 (31.0)	66 (28.2)	140 (30.3)	
Opioid-related variables during pregnancy				
Enrolled in public addiction treatment program for opioid misuse	1268 (27.9)	53 (22.7)	97 (21.0)	.002
OUD diagnosis	3055 (67.1)	104 (44.4)	248 (53.7)	<.001
Overdose event	87 (1.9)	NA^b^	NA^b^	.48
Medication for OUD				
Buprenorphine	1617 (35.5)	NA^b^	96 (20.8)	<.001
Methadone	1265 (27.8)	59 (25.2)	110 (23.8)	
Both	253 (5.6)	NA^b^	22 (4.8)	
None	1416 (31.1)	126 (53.9)	234 (50.7)	
NAS diagnosis	2465 (54.2)	136 (58.1)	288 (62.3)	.002

Abbreviations: 3MD, 3 months before delivery; ED, emergency department; NA, not available; NAS, neonatal abstinence syndrome; OUD, opioid use disorder.

^a^ Among pregnant women who delivered a live infant between October 1, 2011, and December 21, 2015, in Massachusetts.

^b^ Values of fewer than 11 deliveries were not included in accordance with privacy rules.

^c^ At any time from October 1, 2011, to December 31, 2015.

### Medication Use and Type

Overall, 3474 deliveries (66.2%) in our cohort were to women who received any medication for the treatment of OUD in the year before delivery: A total of 1999 women (38.1%) consistently used medication to treat OUD, 1472 women (28.1%) inconsistently used medication to treat OUD, and 1776 women (33.8%) used no medication to treat OUD. Significant differences were observed by racial/ethnic group: 3181 white non-Hispanic women (69.9%), 108 black non-Hispanic women (46.2%), and 228 Hispanic women (49.4%) received any type of medication to treat OUD in the year before delivery, and 1847 white non-Hispanic women (40.6%), 42 black non-Hispanic women (17.9%), and 110 Hispanic women (23.8%) consistently used medication to treat OUD in the 6 months before delivery.

**Figure 2.**
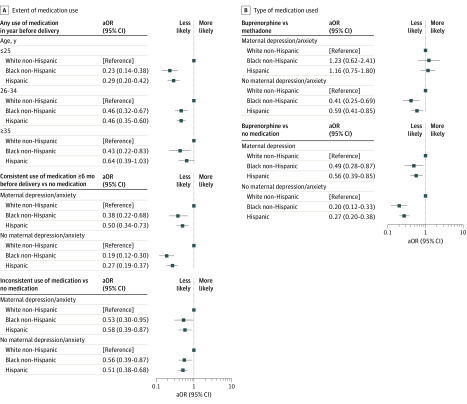
Adjusted Odds Ratios for Extent of Medication Use and Type of Medication Used for Treatment of OUD in Pregnant Women A, Extent of medication use. Adjusted for age, educational level, rural residence, enrollment in Medicaid (MassHealth) during the month of delivery, depression/anxiety diagnosis, emergency department use, and opioid prescription. B, Type of medication used. Adjusted for age, educational level, rural residence, enrollment in Medicaid (MassHealth) during the month of delivery, depression/anxiety diagnosis, emergency department use, and opioid prescription. aOR indicates adjusted odds ratio, and OUD, opioid use disorder.

Table 2 provides details about our unadjusted and adjusted models before stratifying for any positive interactions between covariates and race, and the pseudo-*R*^2^ values for each model. Both black non-Hispanic and Hispanic women had a lower likelihood (adjusted odds ratio [aOR], 0.37; 95% CI, 0.28-0.49 and aOR, 0.42; 95% CI, 0.35-0.52, respectively) of receiving any medication for the treatment of OUD compared with white non-Hispanic women, and this difference increased after stratifying by maternal age at delivery. We identified a significant interaction between race and (1) age for any treatment use and (2) maternal anxiety/depression for the extent of medication use and the type of medication used to treat OUD (Figure 2). Among those 25 years and younger, black non-Hispanic and Hispanic women were 0.23 times (95% CI, 0.14-0.38) and 0.29 times (95% CI, 0.20-0.42) more likely, respectively, to receive any medication for the treatment of OUD compared with white non-Hispanic women. Among women aged 26 to 34 years at delivery, black non-Hispanic and Hispanic women were 0.46 times (95% CI, 0.32-0.67) and 0.46 times (95% CI, 0.35-0.60) more likely, respectively, to receive any medication to treat OUD compared with white non-Hispanic women. Among women 35 years and older, black non-Hispanic and Hispanic women were 0.43 times (95% CI, 0.22-0.83) and 0.64 times (95% CI, 0.39-1.03) more likely, respectively, to receive any medication to treat OUD compared with white non-Hispanic women.

**Table 2.** Adjusted and Unadjusted Odds Ratios for Use of Medication and Type of Medication for Pregnant Women With Opioid Use Disorder

Variable	Odds ratio (95% CI)		Pseudo-*R*^2^	
	Unadjusted	Adjusted^a^	Full model	Model without race/ethnicity
Any treatment use			0.09	0.06
Medication vs no medication				
White non-Hispanic	1 [Reference]	1 [Reference]		
Black non-Hispanic	0.39 (0.30-0.51)	0.37 (0.28-0.49)		
Hispanic	0.44 (0.36-0.53)	0.42 (0.35-0.52)		
Consistency of treatment use			0.09	0.06
Consistent use vs no medication				
White non-Hispanic	1 [Reference]	1 [Reference]		
Black non-Hispanic	0.26 (0.18-0.37)	0.24 (0.17-0.35)		
Hispanic	0.36 (0.28-0.46)	0.34 (0.27-0.44)		
Consistent vs inconsistent treatment use				
White non-Hispanic	1 [Reference]	1 [Reference]		
Black non-Hispanic	0.44 (0.30-0.66)	0.44 (0.30-0.65)		
Hispanic	0.65 (0.50-0.85)	0.64 (0.48-0.83)		
Type of medication			0.12	0.09
Buprenorphine (alone) vs methadone (any)				
White non-Hispanic	1 [Reference]	1 [Reference]		
Black non-Hispanic	0.53 (0.36-0.79)	0.60 (0.40-0.90)		
Hispanic	0.68 (0.52-0.90)	0.77 (0.58-1.01)		
Buprenorphine vs none				
White non-Hispanic	1 [Reference]	1 [Reference]		
Black non-Hispanic	0.27 (0.19-0.39)	0.28 (0.19-0.40)		
Hispanic	0.36 (0.28-0.46)	0.37 (0.29-0.47)		

^a^ Adjusted for age, educational level, rural residence, MassHealth enrollment, depression/anxiety diagnosis, emergency department service use, and opioid prescription during last trimester of pregnancy.

Black non-Hispanic and Hispanic women had a lower likelihood (aOR, 0.24; 95% CI, 0.17-0.35 and aOR, 0.34; 95% CI, 0.27-0.44, respectively) of consistent use of medication for the treatment of OUD compared with white non-Hispanic women. Black non-Hispanic and Hispanic women also had a lower likelihood (aOR, 0.60; 95% CI, 0.40-0.90 and aOR, 0.77; 95% CI, 0.58-1.01, respectively) than white non-Hispanic women of receiving buprenorphine treatment compared with methadone treatment. The results of stratifying by maternal diagnosis of anxiety or depression for both the extent of medication use and the type of medication used to treat OUD revealed that racial and ethnic differences were less substantial (consistent use of medication vs no use of medication to treat OUD and treatment with buprenorphine vs no treatment with medication) or nonsignificant (treatment with buprenorphine vs methadone) for women who had a psychiatric diagnosis (Figure 2). Among those without a diagnosis of anxiety or depression, black non-Hispanic and Hispanic women were 0.38 times (95% CI, 0.22-0.68) and 0.50 times (95% CI, 0.12-0.30) more likely, respectively, to receive consistent medication vs no medication to treat OUD. In addition, black non-Hispanic and Hispanic women with no anxiety or depression were 0.41 times (95% CI, 0.25-0.69) and 0.59 times (95% CI, 0.41-0.85) more likely, respectively, to receive buprenorphine treatment vs methadone treatment compared with white non-Hispanic women. Race/ethnicity explained only 2.7% to 3.0% of the total variance (using the Nagelkerke pseudo-*R*^2^) in our models of the use and type of medication used to treat OUD.

### Sensitivity Analysis

In our first sensitivity analysis, we excluded women identified as having OUD based on an infant diagnosis of NAS alone (n = 766) given the racial/ethnic differences in cohort inclusion based on this variable. The adjusted likelihood of the use of any medication to treat OUD in this group was 42% lower (95% CI, 17%-59%) for black non-Hispanic women and 34% lower (95% CI, 15%-49%) for Hispanic women than for white non-Hispanic women compared with the original cohort, in which the adjusted likelihood was 63% lower (95% CI, 51%-72%) for black non-Hispanic women and 58% lower (95% CI, 48%-65%) for Hispanic women than for white non-Hispanic women. In the second sensitivity analysis, we expanded our sample to include individuals without a clinical indication for medication (n = 5776), and our findings were similar.

The adjusted likelihood of the use of any medication to treat OUD in this larger group was 62% lower (95% CI, 50%-80%) for black non-Hispanic women and 57% lower (95% CI, 48%-64%) for Hispanic women than for white non-Hispanic women compared with the original cohort, in which the adjusted likelihood was 63% lower (95% CI, 51%-72%) for black non-Hispanic women and 58% lower (95% CI, 48%-65%) for Hispanic women. All outcomes and stratified models are available in eTable 6 and eTable 7 in the Supplement. We excluded individuals who received both methadone and buprenorphine therapies (n = 285), and no differences were found in the main models (eTable 8 in the Supplement).

## Discussion

In this study of 5247 pregnant women with OUD who delivered a live infant in Massachusetts, we found that white non-Hispanic women were more likely to have a diagnosis of OUD than black non-Hispanic or Hispanic women. In our sample, the consistent receipt of medication to treat OUD was low among all groups. However, black non-Hispanic and Hispanic women were significantly less likely to receive any medication for the treatment of OUD or to consistently use medication to treat OUD in the 6 months before delivery, with and without adjusting for other maternal characteristics. In addition, among those without depression or anxiety, black non-Hispanic and Hispanic women were significantly less likely to receive buprenorphine treatment compared with methadone treatment or no medication treatment.

Our findings support the analysis by Krans et al,^9^ which indicated that, in a cohort of Medicaid enrollees in Pennsylvania, black non-Hispanic women (27%) and Hispanic women (36%) were less likely than white non-Hispanic women (59%) to receive any medication for the treatment of OUD and were more likely to receive methadone treatment than buprenorphine treatment. Our analyses extended this research by characterizing the extent to which these differences could be associated with race and ethnicity after controlling for other maternal characteristics, and we observed that these disparities were even greater among younger women. Furthermore, we found that black non-Hispanic and Hispanic women were less likely to consistently use medication for the treatment of OUD before delivery, suggesting that not only do disparities exist in treatment initiation but they may also be observed in treatment continuation during pregnancy.

We identified that white non-Hispanic women with OUD were more likely to use buprenorphine compared with black non-Hispanic or Hispanic women, a finding similar to that of the Lagisetty et al^19^ study of buprenorphine prescriptions in a primary care setting. Our analysis, however, was strengthened by accounting for OUD prevalence by racial/ethnic group in our population-based sample. Krawczyk et al^34^ found that black non-Hispanic and Hispanic clients were more likely to access medication for the treatment of OUD from publicly funded treatment programs than were white non-Hispanic clients, but most of these programs dispensed only methadone and had no data available on buprenorphine prescriptions.

Our study benefited from having a robust measure for both buprenorphine and methadone treatments; we found that methadone treatment was similar across all racial/ethnic groups, but we identified a lower use of buprenorphine treatment among black non-Hispanic and Hispanic women without depression or anxiety compared with white non-Hispanic women. These findings are notable given the increase in buprenorphine use during pregnancy since the publication of the Maternal Opioid Treatment: Human Experimental Research (MOTHER) study in 2010, which reported fewer infant opioid withdrawal symptoms with the use of buprenorphine treatment compared with methadone treatment.^35,36,37^ We hypothesize that women with depression/anxiety may have been more engaged in medical or psychiatric care for the treatment of their depression and thus initiated buprenorphine treatment at similar rates, accounting for the similar receipt of office-based buprenorphine treatment among racial/ethnic groups. The reasons underlying inequitable medication use are not well understood, but Hansen and Netherland^38^ and Hansen et al^39^ have suggested that the marketing campaigns of buprenorphine manufacturers have specifically targeted white individuals and that fewer programs and clinicians providing buprenorphine treatment are located in low-income communities of color in New York City.^18,40^

Of importance, race/ethnicity explained only 2.7% to 3.0% of the total variance (using the Nagelkerke pseudo-*R*^2^) in our models of the use and type of medication used to treat OUD, suggesting that many unmeasured factors are associated with treatment engagement and adherence during pregnancy. Pregnancy represents a potentially 9-month opportunity during which frequent engagement with the health care system can support assessment, medication initiation, and continued engagement in services. We identified higher rates of the use of medication to treat OUD during pregnancy than after other high-risk events, such as a single encounter for nonfatal overdose.^41^ However, additional investigation is needed to better understand why one-third of the women with OUD in this cohort were not treated with any medication; further research that includes an examination of maternal age, marital status, insurance status, and geography, which all were associated with differences in the use of medication to treat OUD in our sample, is warranted.

We hypothesize that women’s desire to minimize medication exposures to the fetus and avoid the risk of neonatal withdrawal, shame and stigma because of their drug use, and fear of being reported to child protective services are factors associated with avoiding the use of medication to treat OUD.^42,43,44^ In addition, it is necessary to further elucidate the treatment trajectories of pregnant women with OUD. Lo-Ciganic et al^21^ characterized distinct trajectories of the use of medication to treat OUD, finding that more than 25% of women who initiate treatment report low adherence or early discontinuation.

We hypothesize that a confluence of current and historical factors may be associated with our findings. First, the increasingly punitive policy responses toward pregnant women who use drugs, which were implemented in response to the high rates of cocaine and crack use in the 1980s and 1990s, may make women of color distrustful about disclosing substance use during pregnancy and result in avoidance of treatment.^45,46,47,48^ Next, barriers to the consistent use of medication for the treatment of OUD may include delayed identification of OUD, racial discrimination by clinicians, cultural barriers, perceived stigma, and minimal social supports, all factors associated with low addiction treatment program completion among Hispanic and black non-Hispanic people.^49,50,51,52^ Persistent racial inequities in maternal morbidity and mortality, even after adjusting for other maternal comorbid conditions, suggest that structural racism may be associated with a lower standard of care and fewer treatment options for women of color.^24,53,54^

### Limitations

Our study has several limitations. First, our findings may not be generalizable outside of Massachusetts, a state that provides increased services for pregnant and postpartum women with OUD. Second, race and ethnicity are proxies for a complex number of factors, including not only individual and cultural beliefs but also racism and discrimination. Socioeconomic status is also an important marker of inequity^55,56^; however, household income was not available in our data set, so we included insurance type and educational attainment to estimate socioeconomic status. Third, misclassification bias was possible, as we used a broad definition of OUD that included more factors than insurance claims diagnoses. For example, a woman who had a nonfatal overdose may not have met the *DSM-V* diagnostic criteria for a substance use disorder; thus, the use of medication for the treatment of OUD may not necessarily have been indicated. However, our overall ability to corroborate our definition of OUD across multiple data sources and to include infant diagnosis of NAS to allow for a measure of untreated addiction is a strength of this study. Fourth, we were only able to identify the monthly receipt of buprenorphine treatment and not whether women took the medications they received. Fifth, we did not have information regarding other co-occurring substance use disorders, which may be associated with treatment consistency. Sixth, our data reflect deliveries of infants from 2011 to 2015; changes within the last 5 years, owing to the increasing focus on improving treatment availability for women with OUD, may have occurred.

## Conclusions

Black non-Hispanic and Hispanic women with OUD had significantly lower rates of the use of medication to treat OUD during pregnancy and were less likely to receive buprenorphine treatment compared with white non-Hispanic women. These differences persisted after adjustment for other maternal characteristics. The confluence of disparities in substance use treatment and perinatal care may be associated with even greater inequities in treatment and retention among women of color across the perinatal period. Public health organizations and clinicians caring for women should be aware of potential bias and reduce barriers to using medication for the treatment of OUD among all pregnant and postpartum women to ensure equitable care. Moving forward, an exploration of patient, clinician, hospital, and treatment system characteristics with a consideration of inequities in treatment use will be important to reducing these disparities.

## Notes

Supplement. **eTable 1.** *ICD-9-CM* and *ICD-10-CM* Codes Used for Identification **eTable 2.** Data Sources **eTable 3.** Characteristics of Mothers with Opioid Use Disorder by Inclusion/Exclusion Criteria (N = 5776) **eTable 4.** Characteristics of Mothers With Opioid Use Disorder by Treatment Category During Pregnancy (N = 5247) **eTable 5.** Characteristics of Mothers Receiving Medication for Opioid Use Disorder by Medication Type (n = 3471) **eTable 6.** Unadjusted and Adjusted Odds of Use and Type of Medication for Opioid Use Disorder (Sensitivity Analysis—No NAS Only) **eTable 7.** Unadjusted and Adjusted Odds of Use and Type of Medication for Opioid Use Disorder (Sensitivity Analysis—No Exclusions) **eTable 8.** Sensitivity Analysis #3: Adjusted Odds of Type of Medication for Opioid Use Disorder, Excluding Women Who Received Both Methadone and Buprenorphine (Main Study Cohort, N = 5247)

## References

[1] Haight SC, Ko JY, Tong VT, Bohm MK, Callaghan WM Opioid use disorder documented at delivery hospitalization—United States, 1999-2014. MMWR Morb Mortal Wkly Rep. 2018;67(31):845-849. doi:10.15585/mmwr.mm6731a1 30091969 PMC6089335

[2] American College of Obstetricians and Gynecologists (ACOG) Committee opinion no. 711 summary: opioid use and opioid use disorder in pregnancy. Obstet Gynecol. 2017;130(2):488-489. doi:10.1097/AOG.0000000000002229 28742670

[3] Comer S, Cunningham C, Fishman MJ, *The National Practice Guideline for the Use of Medications in the Treatment of Addiction Involving Opioid Use* American Society of Addiction Medicine; 2015 Accessed May 26, 2019. https://www.asam.org/docs/default-source/practice-support/guidelines-and-consensus-docs/asam-national-practice-guideline-supplement.pdf

[4] American Society of Addiction Medicine *Public Policy Statement on Substance Use, Misuse, and Use Disorders During and Following Pregnancy, With an Emphasis on Opioids Background* American Society of Addiction Medicine; 2017 Accessed May 3, 2018. https://www.asam.org/docs/default-source/public-policy-statements/substance-use-misuse-and-use-disorders-during-and-following-pregnancy.pdf?sfvrsn=644978c2_4

[5] Zedler BK, Mann AL, Kim MM, Buprenorphine compared with methadone to treat pregnant women with opioid use disorder: a systematic review and meta-analysis of safety in the mother, fetus and child. Addiction. 2016;111(12):2115-2128. doi:10.1111/add.13462 27223595 PMC5129590

[6] Mattick RP, Breen C, Kimber J, Davoli M Buprenorphine maintenance versus placebo or methadone maintenance for opioid dependence. Cochrane Database Syst Rev. 2014;16(2):CD002207. doi:10.1002/14651858.CD002207.pub4 15266465

[7] Ordean A, Wong S, Graves L No. 349—substance use in pregnancy. J Obstet Gynaecol Can. 2017;39(10):922-937. doi:10.1016/j.jogc.2017.04.028 28935057

[8] Wolfe EL, Guydish JR, Santos A, Delucchi KL, Gleghorn A Drug treatment utilization before, during and after pregnancy. J Subst Use. 2007;12(1):27-38. doi:10.1080/14659890600823826 22719224 PMC3377323

[9] Krans EE, Kim JY, James AE III, Kelley D, Jarlenski MP Medication-assisted treatment use among pregnant women with opioid use disorder. Obstet Gynecol. 2019;133(5):943-951. doi:10.1097/AOG.0000000000003231 30969219 PMC6483844

[10] Short VL, Hand DJ, MacAfee L, Abatemarco DJ, Terplan M Trends and disparities in receipt of pharmacotherapy among pregnant women in publically funded treatment programs for opioid use disorder in the United States. J Subst Abuse Treat. 2018;89:67-74. doi:10.1016/j.jsat.2018.04.003 29706175

[11] Schiff DM, Nielsen T, Terplan M, Fatal and nonfatal overdose among pregnant and postpartum women in Massachusetts. Obstet Gynecol. 2018;132(2):466-474. doi:10.1097/AOG.0000000000002734 29995730 PMC6060005

[12] Volkow ND, Frieden TR, Hyde PS, Cha SS Medication-assisted therapies—tackling the opioid-overdose epidemic. N Engl J Med. 2014;370(22):2063-2066. doi:10.1056/NEJMp1402780 24758595

[13] Kennedy-Hendricks A, Levin J, Stone E, McGinty EE, Gollust SE, Barry CL News media reporting on medication treatment for opioid use disorder amid the opioid epidemic. Health Aff (Millwood). 2019;38(4):643-651. doi:10.1377/hlthaff.2018.05075 30933576

[14] Volkow ND, Jones EB, Einstein EB, Wargo EM Prevention and treatment of opioid misuse and addiction: a review. JAMA Psychiatry. 2019;76(2):208-216. doi:10.1001/jamapsychiatry.2018.3126 30516809

[15] Olsen Y, Sharfstein JM Confronting the stigma of opioid use disorder—and its treatment. JAMA. 2014;311(14):1393-1394. doi:10.1001/jama.2014.2147 24577059

[16] Gryczynski J, Schwartz RP, Salkever DS, Mitchell SG, Jaffe JH Patterns in admission delays to outpatient methadone treatment in the United States. J Subst Abuse Treat. 2011;41(4):431-439. doi:10.1016/j.jsat.2011.06.005 21821378 PMC3205308

[17] Rosenblum A, Cleland CM, Fong C, Kayman DJ, Tempalski B, Parrino M Distance traveled and cross-state commuting to opioid treatment programs in the United States. J Environ Public Health. 2011;2011:948789. doi:10.1155/2011/948789 21776440 PMC3136171

[18] Hansen HB, Siegel CE, Case BG, Bertollo DN, DiRocco D, Galanter M Variation in use of buprenorphine and methadone treatment by racial, ethnic, and income characteristics of residential social areas in New York City. J Behav Health Serv Res. 2013;40(3):367-377. doi:10.1007/s11414-013-9341-3 23702611 PMC3818282

[19] Lagisetty PA, Ross R, Bohnert A, Clay M, Maust DT Buprenorphine treatment divide by race/ethnicity and payment. JAMA Psychiatry. 2019;76(9):979-981. doi:10.1001/jamapsychiatry.2019.0876 31066881 PMC6506898

[20] Hadland SE, Bagley SM, Rodean J, Receipt of timely addiction treatment and association of early medication treatment with retention in care among youths with opioid use disorder. JAMA Pediatr. 2018;172(11):1029-1037. doi:10.1001/jamapediatrics.2018.2143 30208470 PMC6218311

[21] Lo-Ciganic W-H, Donohue JM, Kim JY, Adherence trajectories of buprenorphine therapy among pregnant women in a large state Medicaid program in the United States. Pharmacoepidemiol Drug Saf. 2019;28(1):80-89. doi:10.1002/pds.4647 30192041 PMC6557135

[22] Le Cook B, McGuire TG, Zuvekas SH Measuring trends in racial/ethnic health care disparities. Med Care Res Rev. 2009;66(1):23-48. doi:10.1177/1077558708323607 18796581 PMC2764787

[23] Smedley BD, Stith AY, Nelson AR, eds; Institute of Medicine (US) Committee on Understanding and Eliminating Racial and Ethnic Disparities in Health Care. *Unequal Treatment: Confronting Racial and Ethnic Disparities in Health Care* National Academies Press; 2003. 25032386

[24] Bryant AS, Worjoloh A, Caughey AB, Washington AE Racial/ethnic disparities in obstetric outcomes and care: prevalence and determinants. Am J Obstet Gynecol. 2010;202(4):335-343. doi:10.1016/j.ajog.2009.10.864 20060513 PMC2847630

[25] Kozhimannil KB, Trinacty CM, Busch AB, Huskamp HA, Adams AS Racial and ethnic disparities in postpartum depression care among low-income women. Psychiatr Serv. 2011;62(6):619-625. doi:10.1176/ps.62.6.pss6206_0619 21632730 PMC3733216

[26] Shavers VL, Shavers BS Racism and health inequity among Americans. J Natl Med Assoc. 2006;98(3):386-396. 16573303 PMC2576116

[27] Williams AR, Nunes EV, Bisaga A, Developing an opioid use disorder treatment cascade: a review of quality measures. J Subst Abuse Treat. 2018;91:57-68. doi:10.1016/j.jsat.2018.06.001 29910015 PMC6039975

[28] Massachusetts Department of Public Health *An Assessment of Opioid-Related Deaths in Massachusetts (2013-2014)* Massachusetts Department of Public Health; 2016 Accessed April 11, 2018. https://www.mass.gov/files/documents/2016/09/pg/chapter-55-report.pdf

[29] An Act Requiring Certain Reports for Opiate Overdoses. Chapter 55, 191st Leg (Mass 2015). August 5, 2015. Accessed October 8, 2017. https://malegislature.gov/Laws/SessionLaws/Acts/2015/Chapter55

[30] Massachusetts Department of Public Health *An Assessment of Fatal and Nonfatal Opioid Overdoses in Massachusetts (2011–2015)* Massachusetts Department of Public Health; 2017 Accessed October 8, 2017. https://www.mass.gov/files/documents/2017/08/31/legislative-report-chapter-55-aug-2017.pdf

[31] Massachusetts Department of Public Health Data Brief: Opioid-Related Overdose Deaths among Massachusetts Residents. Massachusetts Department of Public Health; November 2018 Accessed October 6, 2017. https://www.mass.gov/files/documents/2018/11/16/Opioid-related-Overdose-Deaths-among-MA-Residents-November-2018.pdf

[32] Kotelchuck M An evaluation of the Kessner Adequacy of Prenatal Care Index and a proposed Adequacy of Prenatal Care Utilization Index. Am J Public Health. 1994;84(9):1414-1420. doi:10.2105/AJPH.84.9.1414 8092364 PMC1615177

[33] Nagelkerke NJD A note on a general definition of the coefficient of determination. Biometrika. 1991;78(3):691-692. doi:10.1093/biomet/78.3.691

[34] Krawczyk N, Feder KA, Fingerhood MI, Saloner B Racial and ethnic differences in opioid agonist treatment for opioid use disorder in a U.S. national sample. Drug Alcohol Depend. 2017;178:512-518. doi:10.1016/j.drugalcdep.2017.06.009 28719885 PMC5557040

[35] Jones HE, Kaltenbach K, Heil SH, Neonatal abstinence syndrome after methadone or buprenorphine exposure. N Engl J Med. 2010;363(24):2320-2331. doi:10.1056/NEJMoa1005359 21142534 PMC3073631

[36] Klaman SL, Isaacs K, Leopold A, Treating women who are pregnant and parenting for opioid use disorder and the concurrent care of their infants and children: literature review to support national guidance. J Addict Med. 2017;11(3):178-190. doi:10.1097/ADM.0000000000000308 28406856 PMC5457836

[37] Krans EE, Bogen D, Richardson G, Park SY, Dunn SL, Day N Factors associated with buprenorphine versus methadone use in pregnancy. Subst Abus. 2016;37(4):550-557. doi:10.1080/08897077.2016.1146649 26914546 PMC5596875

[38] Netherland J, Hansen H White opioids: pharmaceutical race and the war on drugs that wasn’t. Biosocieties. 2017;12(2):217-238. doi:10.1057/biosoc.2015.46 28690668 PMC5501419

[39] Hansen H, Netherland J Is the prescription opioid epidemic a white problem? Am J Public Health. 2016;106(12):2127-2129. doi:10.2105/AJPH.2016.303483 27831792 PMC5105018

[40] Hansen H, Siegel C, Wanderling J, DiRocco D Buprenorphine and methadone treatment for opioid dependence by income, ethnicity and race of neighborhoods in New York City. Drug Alcohol Depend. 2016;164:14-21. doi:10.1016/j.drugalcdep.2016.03.028 27179822 PMC5539992

[41] Larochelle MR, Bernson D, Land T, Medication for opioid use disorder after nonfatal opioid overdose and association with mortality: a cohort study. Ann Intern Med. 2018;169(3):137-145. doi:10.7326/M17-3107 29913516 PMC6387681

[42] Cleveland LM, Bonugli R Experiences of mothers of infants with neonatal abstinence syndrome in the neonatal intensive care unit. J Obstet Gynecol Neonatal Nurs. 2014;43(3):318-329. doi:10.1111/1552-6909.12306 24754258

[43] Ostrach B, Leiner C “I didn’t want to be on suboxone at first…”—ambivalence in perinatal substance use treatment. J Addict Med. 2019;13(4):264-271. doi:10.1097/ADM.0000000000000491 30585875

[44] Saia KA, Schiff D, Wachman EM, Caring for pregnant women with opioid use disorder in the USA: expanding and improving treatment. Curr Obstet Gynecol Rep. 2016;5:257-263. doi:10.1007/s13669-016-0168-9 27563497 PMC4981621

[45] Schempf AH, Strobino DM Drug use and limited prenatal care: an examination of responsible barriers. Am J Obstet Gynecol. 2009;200(4):412.e1-412.e10. doi:10.1016/j.ajog.2008.10.055 19217591

[46] Poland ML, Dombrowski MP, Ager JW, Sokol RJ Punishing pregnant drug users: enhancing the flight from care. Drug Alcohol Depend. 1993;31(3):199-203. doi:10.1016/0376-8716(93)90001-7 8462410

[47] Coyer C *Substance Abuse Policies and Prenatal Health Behaviors: Do Punitive Policies Improve Birth Outcomes and Increase Prenatal Care?* Dissertation. Cornell University; 2015 Accessed April 20, 2020. https://paa2015.princeton.edu/papers/153645

[48] Kozhimannil KB, Dowd WN, Ali MM, Novak P, Chen J Substance use disorder treatment admissions and state-level prenatal substance use policies: evidence from a national treatment database. Addict Behav. 2019;90:272-277. doi:10.1016/j.addbeh.2018.11.019 30472535

[49] Saloner B, Le Cook B Blacks and Hispanics are less likely than whites to complete addiction treatment, largely due to socioeconomic factors. Health Aff (Millwood). 2013;32(1):135-145. doi:10.1377/hlthaff.2011.0983 23297281 PMC3570982

[50] Mays VM, Jones AL, Delany-Brumsey A, Coles C, Cochran SD Perceived discrimination in health care and mental health/substance abuse treatment among blacks, Latinos, and whites. Med Care. 2017;55(2):173-181. doi:10.1097/MLR.0000000000000638 27753743 PMC5233585

[51] Pinedo M, Zemore S, Rogers S Understanding barriers to specialty substance abuse treatment among Latinos. J Subst Abuse Treat. 2018;94:1-8. doi:10.1016/j.jsat.2018.08.004 30243409 PMC6157272

[52] Pinedo M, Zemore S, Beltrán-Girón J, Gilbert P, Castro Y Women’s barriers to specialty substance abuse treatment: a qualitative exploration of racial/ethnic differences. J Immigr Minor Health. 2019. Published online September 17, 2019. doi:10.1007/s10903-019-00933-2 31531756 PMC7075735

[53] MacDorman MF, Declercq E, Thoma ME Trends in Texas maternal mortality by maternal age, race/ethnicity, and cause of death, 2006-2015. Birth. 2018;45(2):169-177. doi:10.1111/birt.12330 29314209 PMC5980674

[54] MacDorman MF, Declercq E, Thoma ME Trends in maternal mortality by sociodemographic characteristics and cause of death in 27 states and the District of Columbia. Obstet Gynecol. 2017;129(5):811-818. doi:10.1097/AOG.0000000000001968 28383383 PMC5400697

[55] Williams DR Race/ethnicity and socioeconomic status: measurement and methodological issues. Int J Health Serv. 1996;26(3):483-505. doi:10.2190/U9QT-7B7Y-HQ15-JT14 8840198

[56] Egede LE Race, ethnicity, culture, and disparities in health care. J Gen Intern Med. 2006;21(6):667-669. doi:10.1111/j.1525-1497.2006.0512.x 16808759 PMC1924616

