# Supplementary material for: Assessment of Racial and Ethnic Disparities in the Use of Medication to Treat Opioid Use Disorder Among Pregnant Women in Massachusetts
Source: JAMA Netw Open. 2020 May 26;3(5):e205734. doi: 10.1001/jamanetworkopen.2020.5734 (PMC7251447; doi:10.1001/jamanetworkopen.2020.5734)
Supplement: Supplement. — eTable 1. ICD-9-CM and ICD-10-CM Codes Used for Identification eTable 2. Data Sources eTable 3. Characteristics of Mothers with Opioid Use Disorder by Inclusion/Exclusion Criteria (N = 5776) eTable 4. Characteristics of Mothers With Opioid Use Disorder by Treatment Category During Pregnancy (N = 5247) eTable 5. Characteristics of Mothers Receiving Medication for Opioid Use Disorder by Medication Type (n = 3471) eTable 6. Unadjusted and Adjusted Odds of Use and Type of Medication for Opioid Use Disorder (Sensitivity Analysis—No NAS Only) eTable 7. Unadjusted and Adjusted Odds of Use and Type of Medication for Opioid Use Disorder (Sensitivity Analysis—No Exclusions) eTable 8. Sensitivity Analysis #3: Adjusted Odds of Type of Medication for Opioid Use Disorder, Excluding Women Who Received Both Methadone and Buprenorphine (Main Study Cohort, N = 5247) [file jamanetwopen-3-e205734-s001.pdf]

Supplementary Online Content

Schiff DM, Nielsen T, Hoeppner BB, et al. Assessment of racial and ethnic disparities in the use of medication to treat opioid use disorder among pregnant women in Massachusetts. *JAMA Netw Open*. 2020;3(5):e205734. doi:10.1001/jamanetworkopen.2020.5734

eTable 1. *ICD-9-CM* and *ICD-10-CM* Codes Used for Identification

eTable 2. Data Sources

eTable 3. Characteristics of Mothers with Opioid Use Disorder by Inclusion/Exclusion Criteria (N = 5776)

eTable 4. Characteristics of Mothers with Opioid Use Disorder by Treatment Category During Pregnancy (N = 5247)

eTable 5. Characteristics of Mothers Receiving Medication for Opioid Use Disorder by Medication Type (n = 3471)

eTable 6. Unadjusted and Adjusted Odds of Use and Type of Medication for Opioid Use Disorder (Sensitivity Analysis—No NAS Only)

eTable 7. Unadjusted and Adjusted Odds of Use and Type of Medication for Opioid Use Disorder (Sensitivity Analysis—No Exclusions)

eTable 8. Sensitivity Analysis #3: Adjusted Odds of Type of Medication for Opioid Use Disorder, Excluding Women Who Received Both Methadone and Buprenorphine (Main Study Cohort, N = 5247)

This supplementary material has been provided by the authors to give readers additional information about their work.

© 2020 Schiff DM et al. *JAMA Network Open*

eTable 1. *ICD-9-CM* and *ICD-10-CM* Codes Used for Identification

Characteristic	ICD-9-CM	ICD-10-CM
Opioid Use Disorder (abuse and dependence)	30400-30403, 30470-30473, 30550-30553	F1120, F1121, F1110, F11120-F11122, F11129, F1114, F11150, F11151, F11159, F11181, F11182, F11188, F1119-F11222, F11229, F1123, F1124, F11250, F11251, F11259, F11281, F11282, F11288, F1129, F1190-F11922, F11929, F11913, F11914, F11950, F11951, F11959, F11981, F11982, F11989, F1199
Overdose Encounters	96500-96502, 96509, E8500-E8502	
NAS diagnosis	7795, 76062, 76072	P961, P0449

© 2020 Schiff DM et al. *JAMA Network Open*

eTable 2. Data Sources

A. Contribution of each data source in cohort identification among those included in cohort (n=5,247)

	All	Unique (delivery with only 1 source)
OUD code and/or endocarditis, skin/soft tissue infection, osteomyelitis	3,407 (64.93%)	309 (17.84%)
Overdose code	102 (1.94%)	15 (0.87%)
Publicly-funded opioid tx program enrollment	1,418 (27.02%)	120 (6.93%)
Methadone	1,719 (32.76%)	175 (10.10%)
Buprenorphine	2,037 (38.82%)	347 (20.03%)
NAS	2,889 (55.06%)	766 (44.23%)

B. Number of identifying sources for each delivery in cohort

Total Sources of OUD Identification	
1	1,732
2	1,522
3	1,312
4	552
5 or more	129
**Total**	**5,247**

C. Mean number of identifying sources for each delivery by race/ethnicity

Total Sources by Race/Ethnicity						
	Mean	SD	Median	IQR	Min	Max
WNH	2.3	1.1	2	2	1	6
BNH	1.8	1.1	1	1	1	5
HISP	1.9	1.1	1	2	1	5

OUD=Opioid Use Disorder, Tx = treatment, WNH = white non-Hispanic, BNH = black non-Hispanic, HISP = Hispanic

© 2020 Schiff DM et al. *JAMA Network Open*

eTable 3. Characteristics of Mothers with Opioid Use Disorder by Inclusion/Exclusion Criteria (N = 5776)

	**Included in final sample**		**Excluded from final sample**			
	(n = 5,247)		(n = 529)			
	OUD evidence	NAS Only	Iatrogenic NAS	Chronic Pain	No clinical indication for medication Tx	P-Value
	(n = 4,481)	(n=766)	(n=79)	(n=87)	(n=363)	(5 group)
**Demographics**						
Maternal Age						<0.001
≤ 25 years old	1,247 (27.83%)	243 (31.72%)	19 (24.05%)	*	113 (31.13%)	
26-34 years old	2,674 (59.67%)	378 (49.35%)	38 (48.10%)	47 (54.02%)	204 (56.20%)	
≥ 35 years old	560 (12.50%)	145 (18.93%)	22 (27.85%)	*	46 (12.67%)	
Maternal Race/Ethnicity						<0.001
White non-Hispanic	4,001 (89.29%)	550 (71.80%)	47 (59.49%)	65 (74.71%)	308 (84.85%)	
Black non-Hispanic	159 (3.55%)	75 (9.79%)	13 (16.46%)	*	17 (4.68%)	
Hispanic	321 (7.16%)	141 (18.41%)	19 (24.05%)	*	38 (10.47%)	
Maternal Education						<0.001
High school or less	2,425 (54.12%)	387 (50.52%)	36 (45.57%)	30 (34.48%)	174 (47.93%)	
Some college or more	2,056 (45.88%)	379 (49.48%)	41 (51.90%)	56 (64.37%)	189 (52.07%)	
MassHealth Delivery						<0.001
Yes	4,139 (92.37%)	567 (74.02%)	59 (74.68%)	67 (77.01%)	307 (84.57%)	
No	342 (7.63%)	199 (25.98%)	20 (25.32%)	20 (22.99%)	56 (15.43%)	
Marital Status						<0.001
Married	718 (16.02%)	196 (25.59%)	33 (41.77%)	30 (34.48%)	97 (26.72%)	
Unmarried	3,763 (83.98%)	570 (74.41%)	45 (56.96%)	57 (65.52%)	266 (73.28%)	
Rural/Urban Residence						0.04
Urban	4,001 (89.29%)	709 (92.56%)	*	*	331 (91.18%)	
Rural	480 (10.71%)	57 (7.44%)	*	*	32 (8.82%)	
**Psychosocial Characteristics and Health Care Utilization in Pregnancy**						
ED Visits						<0.001
3 or More	848 (18.92%)	94 (12.27%)	*	13 (14.94%)	41 (11.29%)	
< 3 visits	3,633 (81.08%)	672 (87.73%)	*	74 (85.06%)	322 (88.71%)	
Anxiety Diagnosis						<0.001

© 2020 Schiff DM et al. *JAMA Network Open*

Yes	1,135 (25.33%)	152 (19.84%)	19 (24.05%)	32 (36.78%)	110 (30.30%)	
No	3,346 (74.67%)	614 (80.16%)	60 (75.95%)	55 (63.22%)	253 (69.70%)	
Depression Diagnosis						<0.001
Yes	1,297 (28.94%)	185 (24.15%)	22 (27.85%)	33 (37.93%)	129 (35.54%)	
No	3,184 (71.06%)	581 (75.85%)	57 (72.15%)	54 (62.07%)	234 (64.46%)	
Incarcerated in Prison or Jail†						<0.001
Yes	816 (18.21%)	60 (7.83%)	*	*	39 (10.74%)	
No	3,665 (81.79%)	706 (92.17%)	*	*	324 (89.26%)	
Homelessness†						<0.001
Yes	1,168 (26.07%)	87 (11.36%)	*	*	48 (13.22%)	
No	3,313 (73.93%)	679 (88.64%)	*	*	315 (86.78%)	
Adequacy of Prenatal Care						<0.001
Less than Adequate	1,909 (42.60%)	293 (38.25%)	*	17 (19.54%)	110 (30.30%)	
Adequate	1,208 (26.96%)	221 (28.85%)	*	32 (36.78%)	112 (30.85%)	
Intensive	1,364 (30.44%)	252 (32.90%)	45 (56.96%)	38 (43.68%)	141 (38.84%)	
**Opioid Related Variables in Pregnancy**						
Public Addiction Treatment Program Enrollment for Opioid Problem						<0.001
Yes	1,418 (31.64%)	0 (0.00%)	0 (0.00%)	0 (0.00%)	0 (0.00%)	
No	3,063 (68.36%)	766 (100.00%)	79 (100.00%)	87 (100.00%)	363 (100.00%)	
OUD Diagnosis						<0.001
Yes	3,407 (76.03%)	0 (0.00%)	0 (0.00%)	0 (0.00%)	363 (100.00%)	
No	1,074 (23.97%)	766 (100.00%)	79 (100.00%)	87 (100.00%)	0 (0.00%)	
Overdose Event						<0.001
Yes	102 (2.28%)	0 (0.00%)	0 (0.00%)	0 (0.00%)	0 (0.00%)	
No	4,379 (97.72%)	766 (100.00%)	79 (100.00%)	87 (100.00%)	363 (100.00%)	
Medication for OUD						<0.001
Buprenorphine	1,752 (39.10%)	0 (0.00%)	0 (0.00%)	0 (0.00%)	0 (0.00%)	
Methadone	1,434 (32.00%)	0 (0.00%)	0 (0.00%)	0 (0.00%)	0 (0.00%)	
Both	285 (6.36%)	0 (0.00%)	0 (0.00%)	0 (0.00%)	0 (0.00%)	
None	1,010 (22.54%)	766 (100.00%)	79 (100.00%)	87 (100.00%)	363 (100.00%)	
Any Opioid Prescription (Excluding Bup) (3MB)						<0.001
Yes	183 (4.08%)	0 (0.00%)	*	87 (100.00%)	21 (5.79%)	
No	4,298 (95.92%)	766 (100.00%)	*	0 (0.00%)	342 (94.21%)	

© 2020 Schiff DM et al. *JAMA Network Open*

Infant NAS Diagnosis						<0.001
Yes	2,123 (47.38%)	766 (100.00%)	79 (100.00%)	87 (100.00%)	0 (0.00%)	
No	2,358 (52.62%)	0 (0.00%)	0 (0.00%)	0 (0.00%)	363 (100.00%)	
*Non-zero cells with < 11 deliveries along with next smallest cell have been suppressed in accordance with privacy rules						
†At any time in 2011-2015						
3MB = In the last three months before delivery, ED=Emergency Department; OUD = Opioid Use Disorder; NAS=neonatal abstinence syndrome; Tx=treatment						

© 2020 Schiff DM et al. *JAMA Network Open*

eTable 4. Characteristics of Mothers with Opioid Use Disorder by Treatment Category During Pregnancy (N = 5247)

	Any MOUD Treatment (n=3,471)		≥ 6 Consistent Months (n = 1,999)	Any Other Treatment (n = 1,472)	No Treatment (n = 1,776)	P-Value (2 group)	P-Value (3 group)
**Demographics**							
Maternal Age						<0.001	<0.001
≤ 25 years old	919 (26.48%)		477 (23.86%)	442 (30.03%)	571 (32.15%)		
26-34 years old	2,130 (61.37%)		1,272 (63.63%)	858 (58.29%)	922 (51.91%)		
≥ 35 years old	422 (12.16%)		250 (12.51%)	172 (11.68%)	283 (15.93%)		
Maternal Race/Ethnicity						<0.001	<0.001
White non-Hispanic	3,135 (90.32%)		1,847 (92.40%)	1,288 (87.50%)	1,416 (79.73%)		
Black non-Hispanic	108 (3.11%)		42 (2.10%)	66 (4.48%)	126 (7.09%)		
Hispanic	228 (6.57%)		110 (5.50%)	118 (8.02%)	234 (13.18%)		
Maternal Education						0.870	0.311
High school or less	1,863 (53.67%)		1,095 (54.78%)	768 (52.17%)	949 (53.43%)		
Some college or more	1,608 (46.33%)		904 (45.22%)	704 (47.83%)	827 (46.57%)		
MassHealth Delivery						<0.001	<0.001
Yes	3,231 (93.09%)		1,889 (94.50%)	1,342 (91.17%)	1,475 (83.05%)		
No	240 (6.91%)		110 (5.50%)	130 (8.83%)	301 (16.95%)		
Marital Status						<0.001	<0.001
Married	517 (14.89%)		289 (14.46%)	228 (15.49%)	397 (22.35%)		
Unmarried	2,954 (85.11%)		1,710 (85.54%)	1,244 (84.51%)	1,379 (77.65%)		
Rural/Urban Residence						<0.001	<0.001
Urban	3,078 (88.68%)		1,751 (87.59%)	1,327 (90.15%)	1,632 (91.89%)		
Rural	393 (11.32%)		248 (12.41%)	145 (9.85%)	114 (8.11%)		
**Psychosocial Characteristics and Health Care Utilization in Pregnancy**							
ED Visits						0.012	0.038
3 or More	590 (17.00%)		345 (17.26%)	245 (16.64%)	352 (19.82%)		
< 3 visits	2,881 (83.00%)		1,654 (82.74%)	1,227 (83.36%)	1,424 (80.18%)		
Anxiety Diagnosis						0.819	0.165

© 2020 Schiff DM et al. *JAMA Network Open*

Yes	848 (24.43%)		512 (25.61%)	336 (22.83%)	439 (24.72%)		
No	2,623 (75.57%)		1,487 (74.39%)	1,136 (77.17%)	1,337 (75.28%)		
Depression Diagnosis						0.036	0.109
Yes	948 (27.31%)		548 (27.41%)	400 (27.17%)	534 (30.07%)		
No	2,523 (72.69%)		1,451 (72.59%)	1,072 (72.83%)	1,242 (69.93%)		
Incarcerated in Prison or Jail†						0.197	<0.001
Yes	596 (17.17%)		299 (14.96%)	297 (20.18%)	280 (15.77%)		
No	2,875 (82.83%)		1,700 (85.04%)	1,175 (79.82%)	1,496 (84.23%)		
Homelessness†						0.003	0.003
Yes	873 (25.15%)		480 (24.01%)	393 (26.70%)	382 (21.51%)		
No	2598 (74.85%)		1,519 (75.99%)	1,079 (73.30%)	1,394 (78.49%)		
Adequacy of Prenatal Care						0.115	<0.001
Less than Adequate	1,425 (41.05%)		715 (35.77%)	602 (30.12%)	682 (34.12%)		
Adequate	972 (28.00%)		710 (48.23%)	370 (25.14%)	392 (26.63%)		
Intensive	1,074 (30.94%)		777 (43.75%)	457 (25.73%)	542 (30.52%)		
**Opioid Related Variables in Pregnancy**							
Public Addiction Treatment Program Enrollment for Opioid Problem						<0.001	<0.001
Yes	1,082 (31.17%)		546 (27.31%)	536 (36.41%)	336 (18.92%)		
No	2,389 (68.83%)		1,453 (72.69%)	936 (63.59%)	1,440 (81.08%)		
OUD Diagnosis						<0.001	<0.001
Yes	2,549 (73.44%)		1,547 (77.39%)	1,002 (68.07%)	858 (48.31%)		
No	922 (26.56%)		452 (22.61%)	470 (31.93%)	918 (51.69%)		
Overdose Event						0.344	0.045
Yes	63 (1.82%)		27 (1.35%)	36 (2.45%)	39 (2.20%)		
No	3,408 (98.18%)		1,972 (98.65%)	1,436 (97.55%)	1,737 (97.80%)		
Medication for OUD						<0.001	<0.001
Buprenorphine	1,752 (50.48%)		959 (47.97%)	793 (53.87%)	0 (0.00%)		
Methadone	1,434 (41.31%)		873 (43.67%)	561 (38.11%)	0 (0.00%)		
Both	285 (8.21%)		167 (8.35%)	118 (8.02%)	0 (0.00%)		
None	0 (0.00%)		0 (0.00%)	0 (0.00%)	1,776 (100.00%)		

© 2020 Schiff DM et al. *JAMA Network Open*

Any Opioid Prescription (Excluding Buprenorphine) (3MB)						<0.001	<0.001
Yes	90 (2.59%)		44 (2.20%)	46 (3.13%)	93 (5.24%)		
No	3,381 (97.41%)		1,955 (97.80%)	1,426(96.88%)	1,683 (94.76%)		
Infant NAS Diagnosis						<0.001	<0.001
Yes	1,696 (48.86%)		1,006 (50.33%)	690 (46.88%)	1,193 (67.17%)		
No	1,775 (51.14%)		993 (49.67%)	782 (53.13%)	583 (32.83%)		

† At any time in 2011-2015

3MB= In the last three months before delivery; ED=Emergency Department; OUD = Opioid Use Disorder; NAS=neonatal abstinence syndrome

© 2020 Schiff DM et al. *JAMA Network Open*

eTable 5. Characteristics of Mothers Receiving Medication for Opioid Use Disorder by Medication Type (n = 3471)

	Buprenorphine (n=1,752)	Methadone (w/ both) (n=1,719)	Methadone (w/o both) (n=1,434)	P-Value (3 group)	P-Value (2 group)
**Demographics**					
Maternal Age				0.191	0.066
≤ 25 years old	463 (26.43%)	456 (26.53%)	380 (26.50%)		
26-34 years old	1,054 (60.16%)	1,076 (62.59%)	902 (62.90%)		
≥ 35 years old	235 (13.41%)	187 (10.88%)	152 (10.60%)		
Maternal Race/Ethnicity				0.002	<0.001
White non-Hispanic	1,617 (92.29%)	1,518 (88.31%)	1,265 (88.21%)		
Black non-Hispanic	39 (2.23%)	69 (4.01%)	59 (4.11%)		
Hispanic	96 (5.48%)	132 (7.68%)	110 (7.67%)		
Maternal Education				<0.001	<0.001
High school or less	867 (49.49%)	996 (57.94%)	830 (57.88%)		
Some college or more	885 (50.51%)	723 (42.06%)	604 (42.12%)		
MassHealth Delivery				<0.001	<0.001
Yes	1,541 (87.96%)	1,690 (98.31%)	1,411 (98.40%)		
No	211 (12.04%)	29 (1.69%)	23 (1.60%)		
Marital Status				0.054	0.056
Married	281 (16.04%)	236 (13.73%)	205 (14.30%)		
Unmarried	1,471 (83.96%)	1,483 (86.27%)	1,229 (85.70%)		
Rural/Urban Residence				<0.001	<0.001
Urban	1,507 (86.02%)	1,571 (91.39%)	1,303 (90.86%)		
Rural	245 (13.98%)	148 (8.61%)	131 (9.14%)		
Psychosocial Characteristics and Health Care Utilization in Pregnancy					
ED Visits				<0.001	<0.001
3 or More	251 (14.33%)	339 (19.72%)	277 (19.32%)		
< 3 visits	1,501 (85.67%)	1,380 (80.28%)	1,157 (80.68%)		
Anxiety Diagnosis				<0.001	0.342
Yes	416 (23.74%)	432 (25.13%)	333 (23.22%)		
No	1,336 (76.26%)	1,287 (74.87%)	1,101 (76.78%)		
Depression Diagnosis				0.23	0.423

© 2020 Schiff DM et al. *JAMA Network Open*

Yes	468 (26.71%)	480 (27.92%)	390 (27.20%)		
No	1,284 (73.29%)	1,239 (72.08%)	1,044 (72.80%)		
Incarcerated in Prison or Jail†				<0.001	<0.001
Yes	224 (12.79%)	372 (21.64%)	294 (20.50%)		
No	1,528 (87.21%)	1,347 (78.36%)	1,140 (79.50%)		
Homelessness†				<0.001	<0.001
Yes	327 (18.66%)	546 (31.76%)	433 (30.20%)		
No	1,425 (81.34%)	1,173 (68.24%)	1,001 (69.80%)		
Adequacy of Prenatal Care				<0.001	<0.001
Less than Adequate	652 (37.21%)	773 (44.97%)	630 (43.93%)		
Adequate	556 (31.74%)	416 (24.20%)	353 (24.62%)		
Intensive	544 (31.05%)	530 (30.83%)	451 (31.45%)		
**Opioid Related Variables in Pregnancy**					
Public Addiction Treatment Program Enrollment for Opioid Problem				<0.001	<0.001
Yes	239 (13.64%)	876 (50.96%)	627 (43.72%)		
No	1,513 (86.36%)	843 (49.04%)	807 (56.28%)		
OUD Diagnosis				0.001	0.001
Yes	1,241 (70.83%)	1,308 (76.09%)	1,083 (75.52%)		
No	511 (29.17%)	411 (23.91%)	351 (24.48%)		
Overdose Event				<0.001	<0.001
Yes	15 (0.86%)	48 (2.79%)	36 (2.51%)		
No	1,737 (99.14%)	1,671 (97.21%)	1,398 (97.49%)		
Any Opioid Prescription (Excluding Bup) (3MB)				<0.001	<0.001
Yes	67 (3.82%)	23 (1.34%)	19 (1.32%)		
No	1,685 (96.18%)	1,696 (98.66%)	1,415 (98.68%)		
Infant NAS Diagnosis				<0.001	<0.001
Yes	781 (44.58%)	915 (53.23%)	757 (52.79%)		
No	971 (55.42%)	804 (46.77%)	677 (47.21%)		

† At any time in 2011-2015

3MB= In the last three months before delivery; ED=Emergency Department; OUD = Opioid Use Disorder; NAS=neonatal abstinence syndrome

© 2020 Schiff DM et al. *JAMA Network Open*

eTable 6. Unadjusted and Adjusted Odds of Use and Type of Medication to treat Opioid Use Disorder (Sensitivity Analysis—No NAS Only)

	OR (95% CI)	aOR* (95% CI)	aOR** (95% CI)		Pseudo R^2 Full Model	Pseudo R^2 w/o interaction	Pseudo R^2 - w/o race/ethnicity
**Any Treatment Use**							
White non-Hispanic	1.00 (reference)	1.00 (reference)	1.00 (reference)		0.059	0.059	0.053
Black non-Hispanic	0.59 (0.42-0.82)	0.58 (0.41-0.83)	0.58 (0.41-0.83)				
Hispanic	0.68 (0.53-0.87)	0.66 (0.51-0.85)	0.66 (0.51-0.85)				
**Consistent Use v. None**							
White non-Hispanic	1.00 (reference)	1.00 (reference)	1.00 (reference)		0.061	0.061	0.050
Black non-Hispanic	0.39 (0.26-0.59)	0.38 (0.25-0.58)	0.38 (0.25-0.58)				
Hispanic	0.56 (0.42-0.74)	0.53 (0.40-0.71)	0.53 (0.40-0.71)				
**Consistent Use v. Inconsistent Use**							
White non-Hispanic	1.00 (reference)	1.00 (reference)	1.00 (reference)		0.061	0.061	0.050
Black non-Hispanic	0.44 (0.30-0.66)	0.44 (0.30-0.65)	0.44 (0.30-0.65)				
Hispanic	0.65 (0.50-0.85)	0.63 (0.48-0.82)	0.63 (0.48-0.82)				
**Buprenorphine vs. Any Methadone**							
White non-Hispanic	1.00 (reference)	1.00 (reference)	1.00 (reference)		0.106	0.103	0.096
Black non-Hispanic	0.53 (0.36-0.79)	0.58 (0.39-0.87)	Anx/Dep in Preg	1.20 (0.61-2.37)			
			No Anx/Dep in Preg	0.39 (0.23-0.66)			
Hispanic	0.68 (0.52-0.90)	0.77 (0.58-1.01)	Anx/Dep in Preg	1.13 (0.73-1.76)			
			No Anx/Dep in Preg	0.60 (0.42-0.86)			
**Buprenorphine v. None**							
White non-Hispanic	1.00 (reference)	1.00 (reference)	1.00 (reference)		0.106	0.103	0.096
Black non-Hispanic	0.41 (0.27-0.63)	0.43 (0.28-0.66)	Anx/Dep in Preg	0.63 (0.33-1.20)			
			No Anx/Dep in Preg	0.34 (0.19-0.60)			
Hispanic	0.55 (0.41-0.74)	0.57 (0.42-0.78)	Anx/Dep in Preg	0.85 (0.54-1.35)			
			No Anx/Dep in Preg	0.43 (0.29-0.64)			
**Buprenorphine vs. Exclusively Methadone (excluding individuals receiving both medication types)**							
White non-Hispanic	1.00 (reference)	1.00 (reference)	1.00 (reference)		0.106	0.103	0.096

© 2020 Schiff DM et al. *JAMA Network Open*

Black non-Hispanic	0.52 (0.34-0.78)	0.57 (0.37-0.86)	Anx/Dep in Preg	1.15 (0.56-2.34)			
			No Anx/Dep in Preg	0.39 (0.22-0.66)			
Hispanic	0.68 (0.51-0.91)	0.77 (0.57-1.02)	Anx/Dep in Preg	1.10 (0.69-1.76)			
			No Anx/Dep in Preg	0.61 (0.42-0.88)			
Anx/Dep= Anxiety and/or Depression diagnosis; OR=Odds Ratio							
* Adjusted for age, education, rural residence, MassHealth enrollment, depression/anxiety diagnosis, ED service utilization, and opioid prescription during last trimester of pregnancy							
** Adjusted for age, education, rural residence, MassHealth enrollment, depression/anxiety diagnosis, ED service utilization, opioid prescription during last trimester of pregnancy, and any significant interaction terms							

© 2020 Schiff DM et al. *JAMA Network Open*

eTable 7. Unadjusted and Adjusted Odds of Use and Type of Medication for Opioid Use Disorder (Sensitivity Analysis—No Exclusions)

	OR (95% CI)	aOR* (95% CI)	aOR** (95% CI)		Pseudo R-Square - Full Model	Pseudo R-Square - w/o interaction	Pseudo R-Square - w/o race/ethnicity
**Any Treatment Use**							
White non-Hispanic	1.00 (reference)	1.00 (reference)	1.00 (reference)		0.106	0.101	0.073
Black non-Hispanic	0.41 (0.32-0.52)	0.38 (0.30-0.50)	≤ 25 | Rural	0.80 (0.14-4.51)			
			≤ 25 | Urban	0.21 (0.12-0.35)			
			26-34 | Rural	1.86 (0.30-11.50)			
			26-34 | Urban	0.48 (0.34-0.69)			
			≥ 35 | Rural	1.65 (0.25-11.00)			
			≥ 35 | Urban	0.43 (0.23-0.80)			
Hispanic	0.46 (0.38-0.55)	0.43 (0.36-0.52)	≤ 25 | Rural	0.90 (0.30-2.73)			
			≤ 25 | Urban	0.28 (0.19-0.40)			
			26-34 | Rural	1.51 (0.50-4.58)			
			26-34 | Urban	0.46 (0.36-0.60)			
			≥ 35 | Rural	1.92 (0.59-6.28)			
			≥ 35 | Urban	0.59 (0.37-0.93)			
**Consistent Use v. No Treatment**							
White non-Hispanic	1.00 (reference)	1.00 (reference)	1.00 (reference)		0.101	0.099	0.070
Black non-Hispanic	0.27 (0.19-0.38)	0.25 (0.18-0.36)	Anx/Dep in Preg	0.37 (0.21-0.65)			
			No Anx/Dep in Preg	0.20 (0.13-0.32)			
Hispanic	0.37 (0.30-0.47)	0.35 (0.28-0.44)	Anx/Dep in Preg	0.52 (0.36-0.75)			
			No Anx/Dep in Preg	0.27 (0.20-0.37)			
**Consistent Use v. Inconsistent Use**							
White non-Hispanic	1.00 (reference)	1.00 (reference)	1.00 (reference)		0.101	0.099	0.070
Black non-Hispanic	0.44 (0.30-0.66)	0.44 (0.30-0.66)	Anx/Dep in Preg	0.72 (0.37-1.42)			
			No Anx/Dep in Preg	0.34 (0.21-0.56)			
Hispanic	0.65 (0.50-0.85)	0.64 (0.49-0.84)	Anx/Dep in Preg	0.87 (0.56-1.35)			
			No Anx/Dep in Preg	0.52 (0.37-0.74)			
**Buprenorphine vs. Any Methadone**							

© 2020 Schiff DM et al. *JAMA Network Open*

White non-Hispanic	1.00 (reference)	1.00 (reference)	1.00 (reference)		0.132	0.128	0.104
Black non-Hispanic	0.53 (0.36-0.79)	0.60 (0.40-0.89)	Anx/Dep in Preg	1.23 (0.62-2.41)			
			No Anx/Dep in Preg	0.41 (0.24-0.69)			
Hispanic	0.68 (0.52-0.90)	0.77 (0.58-1.01)	Anx/Dep in Preg	1.15 (0.74-1.78)			
			No Anx/Dep in Preg	0.59 (0.42-0.85)			
			No Anx/Dep in Preg	0.47 (0.36-0.62)			
**Buprenorphine v. None**							
White non-Hispanic	1.00 (reference)	1.00 (reference)	1.00 (reference)		0.132	0.128	0.104
Black non-Hispanic	0.29 (0.20-0.41)	0.29 (0.20-0.41)	Anx/Dep in Preg	0.48 (0.27-0.83)			
			No Anx/Dep in Preg	0.21 (0.13-0.34)			
Hispanic	0.37 (0.29-0.47)	0.38 (0.29-0.48)	Anx/Dep in Preg	0.59 (0.41-0.86)			
			No Anx/Dep in Preg	0.28 (0.20-0.39)			
**Buprenorphine vs. Exclusively Methadone (excluding individuals receiving both medication types)**							
White non-Hispanic	1.00 (reference)	1.00 (reference)	1.00 (reference)		0.129	0.126	0.101
Black non-Hispanic	0.52 (0.34-0.78)	0.58 (0.39-0.89)	Anx/Dep in Preg	1.17 (0.57-2.39)			
			No Anx/Dep in Preg	0.41 (0.24-0.69)			
Hispanic	0.68 (0.51-0.91)	0.77 (0.58-1.02)	Anx/Dep in Preg	1.12 (0.70-1.77)			
			No Anx/Dep in Preg	0.60 (0.42-0.87)			
			No Anx/Dep in Preg	0.28 (0.20-0.39)			
* Adjusted for age, education, rural residence, MassHealth enrollment, depression/anxiety diagnosis, ED service utilization, and opioid prescription during last trimester of pregnancy							
** Adjusted for age, education, rural residence, MassHealth enrollment, depression/anxiety diagnosis, ED service utilization, opioid prescription during last trimester of pregnancy, and any significant interaction terms							

© 2020 Schiff DM et al. *JAMA Network Open*

eTable 8. Sensitivity Analysis #3: Adjusted Odds of Type of Medication for Opioid Use Disorder, Excluding Women Who Received Both Methadone and Buprenorphine (Main Study Cohort, N = 5247)

	OR (95% CI)	aOR* (95% CI)	Pseudo R-Square - Full Model	Pseudo R-Square - w/o interaction	Pseudo R-Square - w/o race/ethnicity
**Buprenorphine vs. Exclusively Methadone (excluding individuals receiving both medication types)**					
White non-Hispanic	1.00 (reference)	1.00 (reference)	0.121	0.116	0.092
Black non-Hispanic	0.52 (0.34-0.78)	0.58 (0.39-0.89)			
Hispanic	0.68 (0.51-0.91)	0.77 (0.58-1.02)			
**Exclusive Methadone vs. None**					
White non-Hispanic	1.00 (reference)	1.00 (reference)	0.121	0.116	0.092
Black non-Hispanic	0.52 (0.38-0.72)	0.48 (0.34-0.66)			
Hispanic	0.53 (0.41-0.67)	0.48 (0.38-0.62)			

© 2020 Schiff DM et al. *JAMA Network Open*
